# The diagnostic value of FNDC5/Irisin in renal Cell Cancer

**DOI:** 10.1590/S1677-5538.IBJU.2017.0404

**Published:** 2018

**Authors:** Diler Us Altay, Esref Edip Keha, Ersagun Karagüzel, Ahmet Menteşe, Serap Ozer Yaman, Ahmet Alver

**Affiliations:** 1 Department of Chemistry and Chemical Processing Technology, Ulubey Vocational School, Laboratory Technology Program, Ordu University, Ordu, Turkey;; 2 Department of Medical Biochemistry, Faculty of Medicine, Karadeniz Technical University Trabzon, Turkey;; 3 Department of Urology, Faculty of Medicine, Karadeniz Technical University Trabzon, Turkey

**Keywords:** Carcinoma, Renal Cell, FNDC5 protein, rat [Supplementary Concept], Urologic Neoplasms

## Abstract

**Purposes:**

The aim of this study was to determine the diagnostic significance of fibronectin type III domain containing protein 5 (FNDC5)/Irisin levels in the sera of patients with renal cell cancer.

**Materials and Methods:**

In the study, 48 individuals were evaluated. The patient group included 23 subjects diagnosed with renal tumor, and the control group of 25 healthy individuals. Patients diagnosed with renal tumor received surgical treatment consisting of radical or partial nephrectomy. Blood specimens were collected and serum FNDC5/Irisin and carcinoembryonic antigen (CEA) levels were determined using enzyme-linked immunosorbent assay (ELISA).

**Results:**

FNDC5/irisin and CEA levels in renal cancer patients were significantly higher compared with the control group (p=0.0001, p=0.009, respectively). Also, FNDC5 levels was more sensitive and specific than CEA levels. The best cut-off points for FNDC5/irisin were >105pg/mL and CEA were >2.67ng/mL for renal cancer.

**Conclusions:**

FNDC5/Irisin may be used as a diagnostic biomarker for renal cancer.

## INTRODUCTION

Type-1 membrane protein FNDC5 contains 212 amino acids (aa). The N-terminal of FNDC5 contains the signal peptide (1-31aa) followed by the “Irisin,” which is 112 amino acids long (32-143aa). The length of the transmembrane domain is 21 amino acids and that of the cytoplasmic domain 48 amino acids (1). FNDC5 is proteolytically cleaved from the N-terminal domain, and a newly identified hormone, irisin, is then formed and released into blood. This hormone is known to act via cell surface receptors, although no such receptor has yet been identified (2). FNDC5 genes are present in humans, mice and rats. Expression of FNDC5 is stimulated by peroxisome proliferator-activated receptor gamma coactivator 1-alpha (PGC1-α), which is a transcriptional co-activator of the peroxisome proliferator-activated receptor gamma nuclear receptor (PPARγ) (3). Serum FNDC5/irisin levels have previously been investigated in obesity, chronic kidney disease, type 2 diabetes mellitus (3-7) and various types of cancer (16-20).

Urological cancers are comprised of bladder, prostate, renal and testis cancers, which are among the 10 most frequent cancers in man except testis cancer. So far, the gold standard diagnosis of urological cancer is pathological diagnosis, and early screening methods are rare. Bladder cancer and kidney cell carcinoma lack specific predictive biomarkers and only some symptoms, for instance, hematuria, might have some effects in finding the existence of cancer (8).

Renal cancers amounts to 2% of the total human cancer burden, with approximately 190.000 new cases diagnosed each year. Although renal tumors can be completely removed surgically, haematogeneous metastasis is frequent and may occur already at an early stage of the disease. Approximately, 85% of renal cancer is renal cell mediated. Renal cell carcinoma is a group of malignancies arising from the epithelium of the renal tubules. The most common type of renal cancer is clear cell renal cell carcinoma, which constitutes 60% to 70% of renal cell carcinomas (9). Clear cell renal cell carcinoma (CCRCC) is the most common type of cancer found in the kidney accounting for ~90% of all kidney cancers. In 2012, there were ~337.000 new cases of RCC diagnosed worldwide with an estimated 143.000 deaths, with the highest incidence and mortality in North America and Europe (10). Several studies have been performed with the aim of developing a biomarker with a high predictive value in renal tumors (11-13).

Carcinoembryonic antigen, first described by Gold and Freedman (1965), is a tumour-associated antigen characterised as a glycoprotein of approximately 180kDa molecular weight. CEA serum levels are known to be elevated in patients with a variety of neoplasms derived from the endoderm and ectoderm. Another studies showed CEA levels increased in renal cancer (11-13).

Based on the objective of developing a biomarker capable of use in renal tumors, we investigated FNDC5/irisin, a marker that has not previously been studied in patients with renal tumor. We compared FNDC5/irisin, with CEA, previously investigated marker in renal tumors.

## MATERIALS AND METHODS

### Study population

This retrospective study involved 23 renal cell cancer patients and 25 healthy controls. Informed consent was obtained from all patients and controls, and approval for the study was given by the local ethics committee of the Karadeniz Technical University Faculty of Medicine. Patients were selected from individuals presenting to the Karadeniz Technical University Medical Faculty Urology clinics. All of the patients were evaluated clinically and they were also previously biochemical and radiologically investigated. Surgical treatment in the form of radical or partial nephrectomy was performed in all cases of diagnosed renal tumor.

Five milliliter (5ml) blood samples for each subject were collected and kept for approximately 30 min in Vacutainer^®^ tubes. These were taken from the peripheral vein and stored at 4°C. Serum specimens were obtained by centrifuging the blood samples at 3000rpm for 10 min. Serum specimens were then stored at -80°C until biochemical analysis.

### Determination of FNDC5/irisin and CEA Levels

FNDC5/irisin levels were determined using an enzyme linked immunosorbent assay (ELISA) kit (USCN, Life Science Inc., Catalog No.USCN-E82576Hu, P.R. China) in line with the manufacturer's instructions. Absorbance of samples was measured at 450nm using a VERSA max tunable microplate reader (designed by Molecular Devices, California, USA). Results were expressed as pg/mL.

### Human (CEA) ELISA Kit

CEA levels were determined using an ELISA kit (Sunred, Ref: DZE201121715, Lot: 201601, Shangai, PRC) in line with the manufacturer's instructions. Absorbance of samples was measured at 450nm using a VERSA max tunable microplate reader (designed by Molecular Devices, California, USA). Results were expressed as ng/mL.

### Statistical Analysis

The test results were analyzed on SPSS (Statistics Program for Social and Science) 13.0.1 (license number: 9069727) statistical software. Data were shown as mean±standard deviation for normal distributed and median (interquartile range) for non-normal distributed variables. The distribution of FNDC5, CEA levels in each group were calculated by Kolmogorov-Smirnov test. Comparisons of the renal cancer's and control groups were done by Student's t-test for normal distribution and by Mann-Whitney U-test for non-normal distribution. Statistical significance was accepted as p<0.05.

## RESULTS

Twenty-three patients were enrolled in the study. The renal tumor group consisted of 17 (73.91%) male and 6 (26.08%) female patients with a mean age of 58.5±15.7 years (range 25 to 80). The healthy control group consisted of 17 (49.1%) male and 8 (50.9%) female, with a mean age of 55.0±13.0 (range 40 to 66).

**Figure 1:**
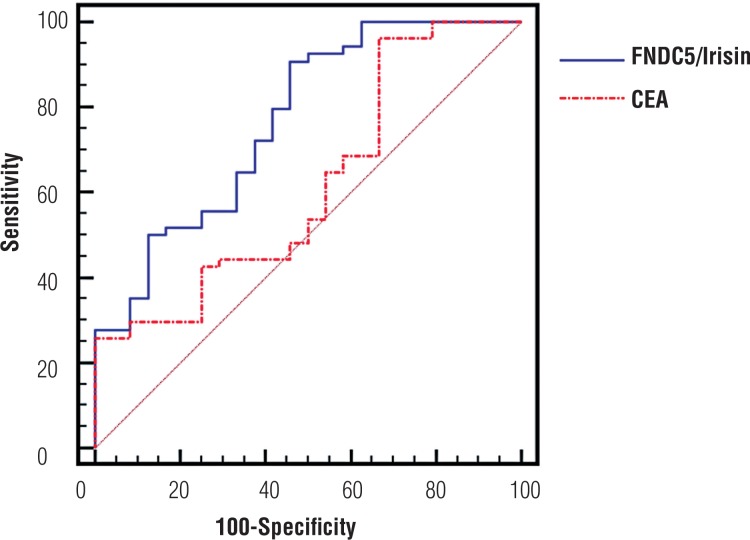
ROC curve analysis of renal cancer patient FNDC5/irisin and CEA values.

**Figure 2:**
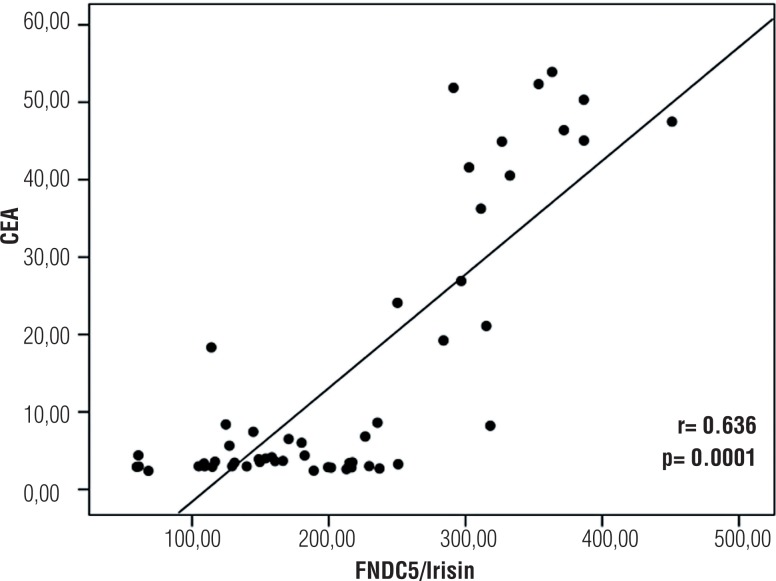
FNDC5/irisin and CEA correlation.

Distribution of biochemical parameters in the renal cancer and control groups is shown in Table-1. Comparison of two groups revealed significantly elevated FNDC5/Irisin levels and CEA in the patients with renal tumor (p=0.0001, p=0.009, respectively). Optimum diagnostic FNDC5/Irisin and CEA cutoff point, AUC according to the receiver operator characteristic (ROC) curve data are shown in Table-2. The pathological distribution of the tumors (pathological type, Fuhrman's nuclear grade, pathological stage) in patients is shown in Table-3. The cases were classified according to the histological type, and clear cell RCC cases were also graded according to the Fuhrman system. There were also no significant difference between groups in terms of pathological type and stage and the Fuhrman's grade (p>0.05). Spearman correlation analysis results of FNDC5/Irisin and CEA in patient, and control groups is shown in Figure-1. In addition, FNDC5 levels showed higher sensitivity and specificity indexes when compared to CEA levels, as observed in Figure-1. There was correlation between biochemical parameters in patient and control group (p=0.0001, r=0.636) (Figure-2).

**Table 1:** FNDC5/irisin and CEA levels.

	Renal Cancer Group (n:23)	Control Group (n:25)	p
**FNDC5/Irisin (pg/mL)**	208±97	110±79	0.0001
**CEA (ng/mL)**	4.08 (2.99-21.9)	3.36 (2.54-5.21)	0.009*

Data were expressed as: mean ± SD, median (inter quarter range for 25-75%)

p shows differences between Control and Cancer according to student t test,

* p shows differences between Control and Cancer according to Mann Whitney U test

**Table 2:** Optimum diagnostic FNDC5/Irisin and CEA cutoff point, AUC according to the receiver operator characteristic (ROC) curve.

Parameters	AUC	95% CI	Cutoff Point	p
FNDC5/Irisin (pg/mL)	0.768	0.658-0.856	>105.2	0.0001
CEA (ng/mL)	0.666	0.558-0.763	>2.67	0.005

**Table 3:** The pathological distribution of the tumors in patients.

		n (%)
**Pathological type**		
	Clear Cell RCC	17 (73.9)
	Papillary RCC	4 (17.3)
	Chromophobe RCC	2 (8.6)
**Fuhrman's nuclear grade**		
	Grade 1	7 (30.4)
	Grade 2	11 (47.8)
	Grade 3	3 (13.0)
	Grade 4	2 (8.6)
**Pathological stage**		
	pT1a	10 (43.4)
	pT1b	7 (30.4)
	pT2a	3 (13.0)
	pT2b	2 (8.6)
	pT3a	1 (4.3)

**RCC** = Renal cell cancer

## DISCUSSION

Substantial promotions have been made in recent years in the diagnosis of renal cancers. But, there is still a need for a marker capable of use in the diagnosis and in determining prognosis of renal cancers.

Several studies have been performed with the aim of developing a biomarker with a high predictive value in renal tumors. Chu et al. found an overall increase of plasma CEA in 56% of the 23 patients studied (11), while Guinan et al. found similar (41%) CEA positivity in their 23 patients with renal-cell carcinoma (12). Cases et al. (1991) showed that CEA, CA-50 and CA-125 levels were elevated in serum of patients with chronic renal failure and in haemodialysis patients (13). Karaguzel et al. showed that signal peptide, CUB domain and EGF like domain containing 1 (SCUBE-1) appears to represent a promising bio-marker in the diagnosis and follow-up of cases of renal tumor (14).

FNDC5 is a type-1 membrane protein. Potential roles and applications of serum FNDC5/irisin in obesity, chronic kidney disease, Type 2 diabetes mellitus have been investigated in previous studies (3-7).

Recently, some researchers want to reveal cancer and irisin relationship. Moon et al. showed that physiological (5-10nmol/L) and physiologically/pharmacologically high concentrations (50-100nmol/L) of irisin had no in vitro effect on cell proliferation and malignancy potential of obesity-related cancer cell lines (15). Us Altay et al. study is about irisin levels in gastric cancer in mice. They revealed that irisin levels increase in the circulation with the development of gastric cancer (16). Increased irisin immunoreactivity in tissues obtained from breast, ovary, cervix carcinomas, and endometrial hyperplasia suggest critical role of this peptide during carcinogenesis (17). Provatopoulou et al. aimed to examine the association between irisin and breast cancer and to evaluate the ability of serum irisin levels to discriminate between breast cancer patients and controls. Serum levels of irisin were significantly lower in breast cancer patients compared to controls (18). Irisin is a protein involved in heat production by converting white into brown adipose tissue, but there is no information about how its expression changes in cancerous tissues. In Aydın et al. study, they used irisin antibody immunohistochemistry to investigate changes in irisin expression in gastrointestinal cancers compared to normal tissues. Histoscores (area intensity) indicated that irisin was increased significantly in gastrointestinal cancer tissues, except liver cancers (19). Gaggini et al. showed that in human hepatocellular carcinoma FNDC5/irisin expression increased (20). Shoa et al. showed that irisin suppresses the migration, proliferation, and invasion of lung cancer cells via inhibition of epithelial-to-mesenchymal transition (21). In our research renal cancer patients FNDC5/irisin and CEA levels were significantly higher compared with the control group. Furthermore, FNDC5 is more sensitive and specific than previously investigated marker in renal tumors, CEA.

The major limitation of our study is the relatively small number of patients and controls involved. Also, no demographic values and routine laboratory findings were given to groups, because our study was for diagnostic marker research so no need to use routine laboratory findings. Only the parameters age and gender numbers were evaluated and there were nearly the same.

Our study was the first that evaluated irisin in renal cell cancer and irisin levels increased significantly. But what amount of increase irisin level in renal cell cancers yet we don't know. Oxidative stress markers (for lipid, protein, DNA oxidations) and inflammation markers must be searched. New studies are been planned to lighten the pathways.

## LIMITATIONS

The major limitation of the study is the relatively small number of patients and controls involved. However, in terms of the novel idea that FNDC5 is a diagnostic biomarker, our study can be considered pioneering research in the field and can serve as a basis for further comprehensive studies.

## ETHICAL APPROVAL

Approval for the study was given by the Local Ethical Committee under reference no. 2014-16.
